# Supplementary material for: Tead1a Initiates Transcriptional Priming Through the TEAD1a/YAP‐Notch1‐Spi1/Cebpα Axis to Promote Neutrophil Fate
Source: Adv Sci (Weinh). 2025 Aug 28;12(41):e05441. doi: 10.1002/advs.202505441 (PMC12591127; doi:10.1002/advs.202505441)
Supplement: Supplementary file 4 — Supplementary Dataset [file ADVS-12-e05441-s004.zip › 3-5.zip!/3-5/02-5.cluster/cluster12/Enrich/ego_all.pdf]

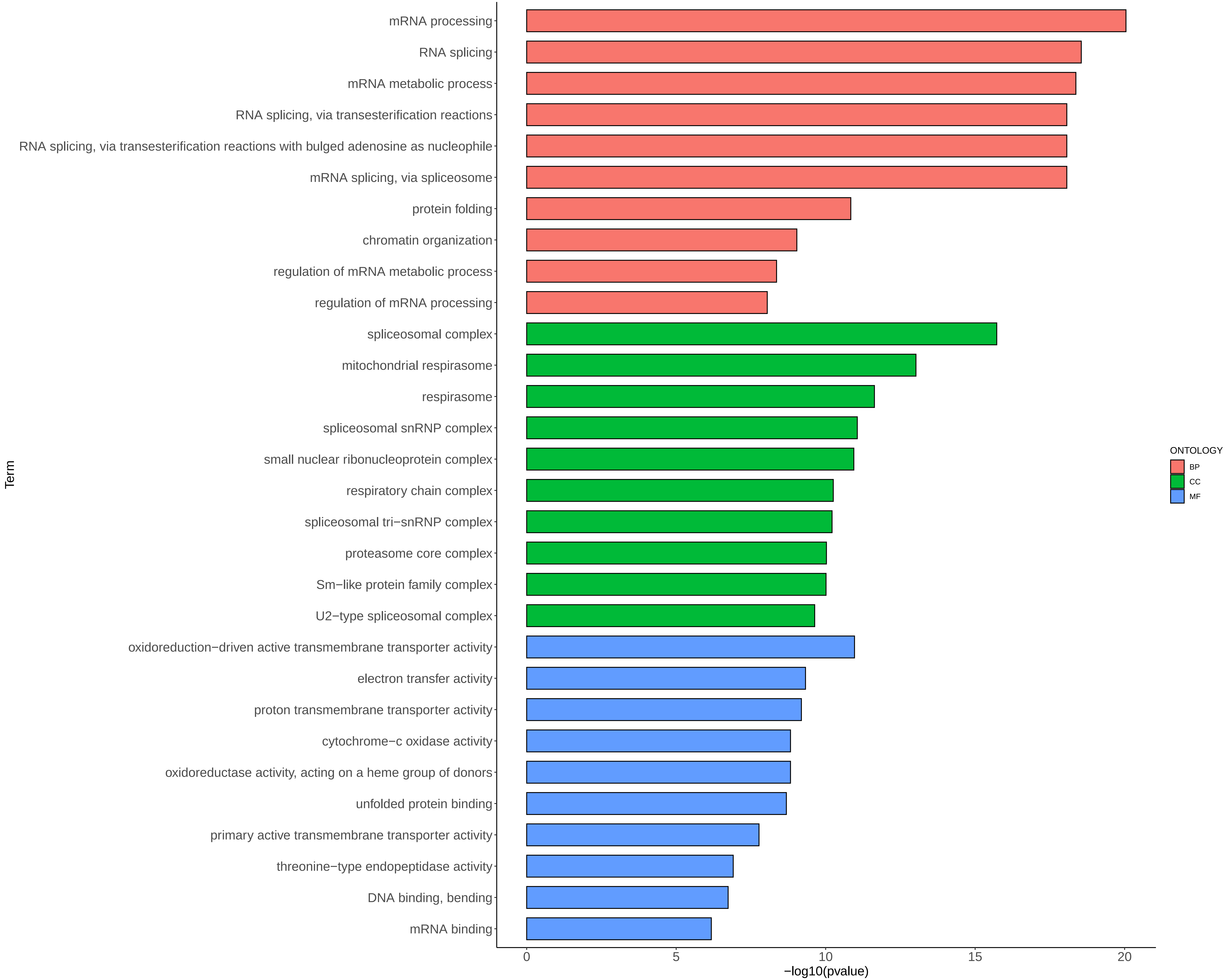
mRNA processing
RNA splicing
mRNA metabolic process
RNA splicing, via transesterification reactions
RNA splicing, via transesterification reactions with bulged adenosine as nucleophile
mRNA splicing, via spliceosome
protein folding
chromatin organization
regulation of mRNA metabolic process
regulation of mRNA processing
spliceosomal complex
mitochondrial respirasome
respirasome
spliceosomal snRNP complex
small nuclear ribonucleoprotein complex
respiratory chain complex
spliceosomal tri−snRNP complex
proteasome core complex
Sm−like protein family complex
U2−type spliceosomal complex
oxidoreduction−driven active transmembrane transporter activity
electron transfer activity
proton transmembrane transporter activity
cytochrome−c oxidase activity
oxidoreductase activity, acting on a heme group of donors
unfolded protein binding
primary active transmembrane transporter activity
threonine−type endopeptidase activity
DNA binding, bending
mRNA binding
Term
0
5
10
15
20
−log10(pvalue)
ONTOLOGY
BP
CC
MF
